# Understanding Adolescents’ Perceptions and Aspirations Towards Their Relationship With Personal Technology: Survey Study

**DOI:** 10.2196/27852

**Published:** 2021-12-23

**Authors:** Muhammad Jee, Alsa Khan, Nazneen Nazneen

**Affiliations:** 1 Twinbits Flower Mound, TX United States; 2 Userwise Consulting Mountain View, CA United States

**Keywords:** adolescents’ perceptions, personal technology, technology relationship, adolescents as technology users, adolescents as technology bystanders

## Abstract

**Background:**

Understanding adolescents' relationship with technology is a pressing topic in this digital era. There seem to be both beneficial and detrimental implications that originate from use of technology by adolescents. Approximately 95% of adolescents have access to a smartphone, and several studies show a positive correlation between screen addiction and trends of anxiety and depression. At the same time, research shows that two-thirds of adolescents believe that technology is a necessity for connecting and making new friends.

**Objective:**

The aim of this formative study was to understand adolescents' perception of their own and others’ relationship with personal technology.

**Methods:**

A survey was conducted with 619 adolescents ranging in age from 13 to 19 years. Adolescents were asked how they perceived the relationship with their personal technology, how they perceived others' (parents, siblings, or friends) relationship with personal technology, and how they wish to relate to their personal technology in the future.

**Results:**

"Essential,” “Distractive,” and “Addictive” were the most commonly selected descriptors to describe both adolescents' own relationship with technology (essential: 106/619, 17.1%; distractive: 105/619, 17%; addictive: 88/619, 14.2% ) and others’ relationship as well (essential: 96/619, 15.6%; distractive: 88/619, 14.3%; addictive: 90/619, 14.5%). Adolescents selected “Provides an escape” more to describe their own relationship with technology. Whereas, they selected “It's just a tool” and “Creates Barrier” more to describe others' relationship with technology. These trends are consistent across ages and genders. In addition, adolescents' aspirations for their relationship with their personal technology varied across ages: 13 to 15-year olds' top choice was “best friend”, 16 to 17-year olds’ top choice was “I don't believe in personal connection with mobile technology,” and 18 to 19-year olds’ top choice was “My personal assistant.”

**Conclusions:**

Our 3-lens method allows us to examine how adolescents perceive their relationship with personal technology in comparison to others, as well as their future technological aspirations. Our findings suggest that adolescents see both communalities as well as differences in their own and others' relationships with technology. Their future aspirations for personal technology vary across age and gender. These preliminary findings will be examined further in our follow-up research.

## Introduction

### Background

As personal technology is advancing rapidly, adolescents’ technology adoption and usage are continuously on the rise. Smartphone use in the adolescent population is nearly universal. Approximately 95% of adolescents in the USA say they have a smartphone or have access to one [1].

Smartphones provide a number of benefits, including increased productivity, efficient information seeking, and enhanced access to meaningful support networks during times of distress and ongoing illnesses [2-4]. Nearly two-thirds of the US adolescent population report that personal technology and social media enable them to make new friends and allow them to bond with people [5]. Youth social media use is also related to an increase in empathy; both in their ability to understand (cognitive empathy) and share the feelings of peers (affective empathy) [6].

Despite these benefits, an increasing amount of studies have revealed the adverse effects associated with adolescents' hyperconnected relationship with personal technology. Consequences of excessive technology dependency include lower self-motivation, decreased social skills, screen addiction, and mental health problems such as anxiety and depression [1,7-9].

With technology having both positive and negative implications on adolescents, an in-depth understanding of adolescents' relationship with technology is necessary.

Research shows that due to unique social, cultural, and developmental factors, adolescents' technology use behaviors differ from those of adults [10-13]. Thus, adolescents' perceptions and choices about technology may not be identical to adults’ and are worth investigating.

The goal of our study is to explore how adolescents perceive their own and others' relationship with technology. Understanding and acknowledging independent perceptions about personal technology is critical for informing technology use management, policies, design, health interventions, teaching, and parenting that resonate with adolescents’ attitudes.

The key contribution of this paper is our holistic approach that interprets adolescents’ relationship with personal technology via 3 lenses. Lens 1 is adolescents' perception of their current relationship with technology as users. Lens 2 is adolescents' perception of others' (parents, siblings, or friends) relationship with technology as bystanders. Lens 3 is adolescents’ aspiration of what relationship, if any, they wish to have with their personal technology in the future. Unlike past research, our approach not only characterizes adolescents' perceptions of their own relationship with technology, but it also frames how adolescents perceive others' relationships with technology. Even though a few studies have also considered adolescents' perception of their parents' or peers’ use of technology [14,15], our study examines a broader trusted social circle of parents, siblings, or friends. This 3-lens approach is a holistic method as it not only reflects how adolescents relate to technology presently but also covers future aspirations of how adolescents wish to relate to technology. Our 3-lens approach allows for an overarching comparison of adolescents’ perception about relationship with technology as users versus as bystanders, as well as their current versus aspirational perspectives. Hence, our research identifies the similarities and differences in how adolescents perceive their own and others' relationships with technology.

In sum, this paper explores these open questions: How do adolescents perceive others' (parents, siblings, or friends) relationship with personal technology and how does it compare from theirs? What relationship, if any, do adolescents wish to have with their personal technology in the future and how does it relate to their perception of their present relationship with technology?

### Prior Work

#### Psychological and Behavioral Implications of Technology

The ubiquity of the smartphone has led researchers to devote much attention to the psychological, behavioral, and social implications of personal technologies. There is vast research into the benefits of social media: sustaining close friendships, building new connections with individuals from diverse backgrounds, demonstrating support for meaningful causes, and becoming civic minded [16,17]. Moreover, smartphone-based interventions enhance the effects of policies on a range of outcomes, including the adoption of positive healthy habits and educational activities [18,19]. Smartphone use seems to have significant impacts on improving student performance, teaching, and learning experiences and is regarded as a key component in the development of social environment [20]. Students benefit from the incorporation of smartphone use in educational activities by efficiently accessing vast course content, participating in debate sessions with professors, and retrieving information regarding student performance [21,22].

Young adults' excessive, addictive, and problematic technology use has been continuously reported alongside the rise in technology adoption. An abundance of research has correlated smartphone usage with negative psychological and behavioral implications, including anxiety, insomnia, and depression [23,24]. Constantly checking communication updates, feeling restless without close proximity to a phone, and suffering delays in professional performance due to prolonged phone activities are indicators of smartphone addiction [25,26]. Excessive use of smartphones can also result in various impacts on physical health, such as fatigue, indigestion, sleep issues, and eyesight problems [27,28]. Parasuraman et al [29] investigated the impact on daily life of smartphones on 55-year-old and 18-year-old age groups and learned that a significant portion of the participants had an addiction to smartphone usage but were unaware of it due to smartphones having become an integral part of their lives [29].

Adolescents who are addicted to personal technology face diminishing social skills and challenges in developing friendships in the real world [30]. Exploring the relationship between social internet use and loneliness, Nowland et al [31] concluded that loneliness can be reduced when the digital world is used to maintain or forge more social connections. However, when people use the internet to avoid physical social activities and day-to-day problems, their loneliness increases. Consequently, they develop a preference to continue using the internet in a way that displaces time spent in offline social interactions. One study demonstrated that adolescents who were more addicted to their phones had an increased risk of feeling the 6 variants of social alienation: powerlessness, normlessness, meaninglessness, self-estrangement, cultural estrangement, and social isolation [32].

#### Correlation of Age and Gender With Technology Usage

Many studies have also assessed the potential correlation of age, gender, and personality variables on smartphone addiction. Adolescents with somatization, poor self-control, interpersonal sensitivity, and hostility tend to be more likely to get addicted to smartphones [33-35]. Neuroticism, conscientiousness, and openness are traits that were found to be negatively correlated with smartphone addiction [36]. Furthermore, research shows that males and females use their phones for different reasons. Female smartphone use is more strongly related to sociability, interpersonal relationships, and the desire to maintain connections [7]. Comparatively, males use their smartphones more extensively for media sharing, video games, and online searches [36]. Many studies found smartphone addiction being more prevalent in younger adolescents [37,38]. A study involving 1529 students aged 11 to 18 years found that younger adolescents (11-14 years) had a higher prevalence of problematic smartphone usage than did older adolescents (15-18 years) [15].

#### Perceptions About Self and Others’ Technology Usage

When US adolescents were asked directly, 31% perceived the effects of social media as mostly positive, 45% believed the effects to be neither positive nor negative, and 24% stated the effects as mostly negative. Those who considered the effects of social media to be positive indicated that it helps in maintaining connectivity with family and friends, obtaining access to information, and meeting like-minded people. Those who considered the effects of social media to be negative explained that it increases the risks of addiction, hate speech, neglecting face-to-face contacts, and obtaining unrealistic views of others’ lives [39]. Similarly, Ozkan and Solmaz [40] examined the correlation between mobile addiction and perception of one’s own personal technology. Through a survey of 18 to 23-year-old university students, they concluded that addiction to technology is related to self-perception technology use. There was a statistically significant correlation between the perception that smartphone apps are useful tools for communicating with people and time being spent on the phone.

Davis and Dinhopl [14] studied the similarities and differences in the way parents and adolescents described their own and each other’s phone use in the context of family life. Both expressed a lack of agency in their own and each other’s smartphone use, feeling displaced by the other’s smartphone and highly reliant on their own smartphone. In addition, parents felt guilty about the impact of their phone overuse on their children, whereas adolescents’ expression of guilt was based on what their parents and society thought of their phone use. Lopez-Fernandez et al [15] conducted a study in which adolescents were asked to indicate whether they felt that any of their peers used their smartphones excessively. Results showed that adolescents with problematic smartphone usage were more likely to consider their peers’ smartphone usage to be problematic.

Previous research has investigated adolescents’ relationship with technology through their use of technology, their own perceptions, or their parents’ perspectives. Whereas, our research used a multi-lens approach to explore alignments and mismatches in adolescent’s perceptions of their own versus others’ relationship with technology as well as their current versus aspirational relationship.

## Methods

### Participants

A total of 619 adolescents participated in the online survey. Among the participants, 58.8% (364/619) were females, while 39.9% (247/619) were males. Table 1 shows participants’ demographics.

From March 2020 to April 2020, this online survey was deployed through different social media platforms to a diverse range of adolescents. Participation in the survey was voluntary, and no incentives were offered. In addition, no identifiable data were collected.

**Table 1:** Participants’ demographics (N=619).

Demographics		Segment size, n (%)
**Gender**		
	Male	247 (39.90%)
	Female	364 (58.80%)
	Prefer not to specify	8 (1.29%)
**Age**		
	13-15 years	76 (12.28%)
	16-17 years	410 (66.24%)
	18-19 years	133 (21.49%)

### Three-Lens Approach

**Figure 1:**
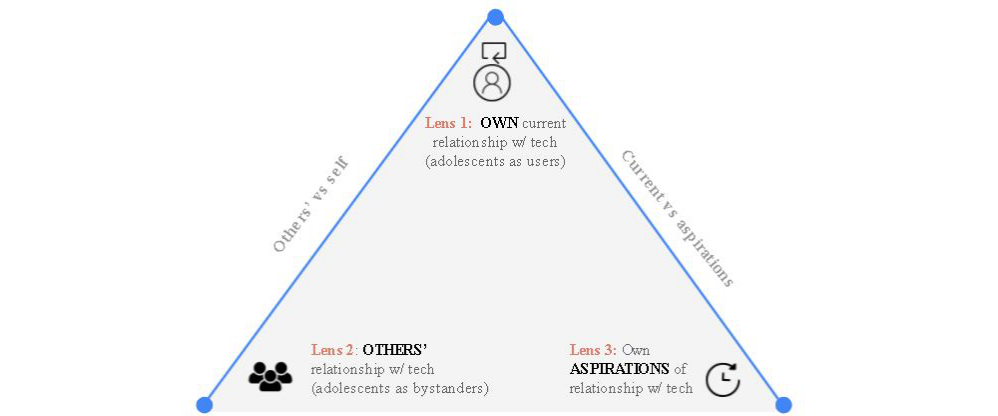
A model illustrating our 3-lens approach.

The survey included 3 main questions representing 3 lenses to understand how adolescents relate to technology. These questions are designed to analyze adolescents’ perceptions of their own personal technology (lens 1: adolescents as users) versus the opinion of adolescents of their parents, siblings, and friends' use (lens 2: adolescents as bystanders) and to identify the type of relationship they desire to have with their technology (lens 3: aspirational). Figure 1 illustrates our novel 3-lens approach.

By bringing all 3 lenses together, we captured a more holistic understanding of adolescents’ perception of their own relationship with technology in comparison with both adolescents’ perceptions of others' relationship with technology as well as adolescents’ aspirations of their technology relationship. Table 2 lists all 3 questions along with options presented in the survey.

**Table 2:** List of all 3 questions along with options presented in the survey.

Question	Options
Q1: Choose 3 options that best describe the relationship between you and your personal technology.	Options: Creates barrier, Enabler, Overwhelming, It’s just a tool, Empowering, Private, Addictive, Essential, Distractive, Hurtful, Provides an escape, Emotional, Other
Q2: Choose 3 options that you think best describe the relationship between your parents, siblings, or friends and their personal technology.	Options: Creates barrier, Enabler, Overwhelming, It's just a tool, Empowering, Private, Addictive, Essential, Distractive, Hurtful, Provides an escape, Emotional, Other
Q3: What persona would you wish your personal technology to match the most with?	Options: I don't believe in personal connection with mobile technology, My best friend, My twin sibling, My personal assistant, My coach/mentor, Other

### Descriptor Selection Process

In question 1, out of 12 descriptors, respondents were asked to choose 3 descriptors that best describes their relationship with their personal technology. Similarly, in question 2, from the same 12 descriptors, adolescents were asked to choose 3 that best described their perception of the relationship of their parents, siblings, or friends with their personal mobile technology.

For context, 2 of the 3 authors (AK and MH), who are high school students and adolescents, had firsthand experience learning from their peers and immersing in the high school environment and other adolescents' online social networks. The level of communication, comfort, and openness at the peer level, provided a unique platform to this research.

In round 1, an initial list of descriptors for questions 1 and 2 was compiled based on learning from unstructured observations and semistructured interviews of 5 high school peers. In the interviews, the adolescents were asked an open-ended question to identify descriptors they would use to describe relationships with personal technology. The descriptors that were mentioned were “Empowering,” “Provides an escape,” “It’s just a tool,” and “Enabler.” These 4 words were used as a general guideline to develop a larger list of descriptors.

In round 2, we added 8 additional descriptors to the initial list by further learning from literature reviews related to adolescents' usage of technology, and personality and profile assessments like the Activity Vector Analysis and Adjective Check List [3-5,7-9,23,24,29,32,41-43]. Out of a list of positive and negative adjectives, these profile assessments allow individuals to choose adjectives that best describe themselves and others’ perception of them.

A list of 12 descriptors was finalized by ensuring that descriptors fall within a spectrum of positive and negative for a diverse understanding of adolescents and technology relationships. After the list was completed, we conducted another round of semistructured interviews with a new sample of 4 high school adolescents to judge each descriptor as a “positive,” “neutral,” and “negative” relationship with personal technology. The descriptors provided in the survey as response to questions 1 and 2 were categorized by words ranging from those that describe the strongest positive relationships (Essential, Empowering, Enabler, Private, Emotional), the neutral relationships (It’s just a tool), and the strongest negative relationships (Provides an escape, Distractive, Creates Barrier, Overwhelming, Hurtful).

Options for question 3, in which adolescents were asked which persona they would wish for in their personal technology, were based on trusted relationships (eg, best friends, siblings), exciting technology offerings (eg, personal assistant Siri, Alexa, Google Assistant), and coach or mentors (eg, learning and fitness apps). The option “I don't believe in personal connection with mobile technology” was included for completeness. Selections for all 3 questions were randomized to eliminate any order effect.

## Results

### Analysis

This section summarizes analysis of key responses within individual lenses.

#### Adolescents' Perception of Their Own Relationship With Their Personal Technology as Users

The top 3 selected descriptors that adolescents chose to describe their own relationship with technology were “Distractive,” “Essential,” and “Provides an escape.” This trend was consistent across genders and age groups (13 to 15-, 16 to 17-, and 18 to 19-year olds).

However, “Distractive” was chosen more often by females. In addition, 13 to 15-year olds selected “Provides an escape” the most. Among all descriptors, “Hurtful” was the least selected across all ages and genders. Table 3 summarizes the percentage of times each descriptor was selected overall (by everyone) and within each segment (male, female, 13 to 15-year old, 16 to 17- year old, 18 to 19-year old).

**Table 3:** Table of the percentage of times each descriptor was selected for describing adolescents’ own relationship with technology. Sorted in descending order of overall percentages. Absolute values are not provided as percentages have been adjusted by weighting. Participants were asked to choose 3 choices to describe their own relationship with technology; the values are normalized to sum up to 100%.

Descriptors in Q1	Overall	Male	Female	Age 13-15	Age 16-17	Age 18-19
Distractive	17%	14%	20%	14%	18%	17%
Essential	17%	17%	17%	14%	18%	18%
Provides an escape	16%	15%	17%	19%	16%	15%
Addictive	14%	13%	15%	14%	14%	14%
Private	8%	8%	8%	10%	8%	7%
It's just a tool	8%	11%	5%	8%	7%	9%
Empowering	7%	8%	6%	6%	6%	7%
Enabler	5%	7%	3%	2%	5%	5%
Other	4%	5%	2%	11%	2%	4%
Overwhelming	3%	2%	4%	4%	3%	4%
Emotional	3%	3%	3%	4%	3%	2%
Creates barrier	2%	2%	2%	2%	2%	2%
Hurtful	1%	0%	1%	1%	1%	1%

#### Adolescents’ Perception of Others’ Relationship With Personal Technology as Bystanders

To describe others' (parents, siblings, friends) relationship with technology, the top 3 descriptors selected by adolescents were “Essential,” “Distractive,” and “Addictive.” Among all descriptors, “Hurtful” was least selected. These trends were consistent across genders and age groups. Table 4 summarizes the percentage of times each descriptor was selected.

**Table 4:** Table of the percentage of times each descriptor was selected for describing others' relationship with technology. Sorted in descending order of overall percentages. Absolute values are not provided as percentages have been adjusted by weighting. Participants were asked to choose 3 choices to describe their own relationship with technology; the values are normalized to sum up to 100%.

Descriptors in Q2	Overall	Male	Female	Age: 13-15	Age: 16-17	Age: 18-19
Essential	16%	15%	16%	14%	16%	15%
Distractive	14%	14%	15%	13%	14%	15%
Addictive	14%	13%	15%	13%	15%	15%
It’s just a tool	13%	13%	13%	16%	13%	13%
Private	9%	9%	8%	8%	9%	8%
Provides an escape	8%	9%	7%	7%	7%	9%
Creates Barrier	7%	6%	7%	5%	8%	5%
Empowering	5%	6%	4%	7%	4%	5%
Enabler	5%	5%	5%	6%	6%	4%
Overwhelming	4%	4%	4%	4%	4%	4%
Emotional	3%	4%	3%	4%	3%	4%
Hurtful	2%	2%	2%	3%	2%	2%
Other	2%	1%	1%	5%	1%	2%

#### Adolescents’ Aspirations of a Persona They Wish for Their Personal Technology

Adolescents’ aspirations of a persona for their personal technology varied across age ranges. The majority (39/74, 52%) of females in the 13 to 15-year old group selected “My best friend” the most. The 16 to 17-year-old group selected “I don't believe in personal connection with mobile technology” and “My personal assistant”, whereas the 18 to 19-year-old group chose “My personal assistant” most frequently. Table 5 shows the percentage of times each persona was selected.

**Table 5:** Table of the percentage of times each persona was selected as adolescents' aspiration of their personal technology.

Q3	Overall, n/N (%)	Age 13-15, n/N (%)	Age 16-17, n/N (%)	Age 18-19, n/N (%)
My personal assistant	208/619 (33.6)	11/76 (14.5)	136/410 (33.2)	61/133 (45.9)
I don't believe in personal connection with mobile technology	199/619 (32.1)	24/76 (31.6)	141/410 (34.4)	34/133 (25.5)
My best friend	148/619 (23.9)	31/76 (40.8)	100/410 (24.4)	17/133 (12.8)
My coach/mentor	34/619 (5.5)	6/76 (7.9)	16/410 (3.9)	12/133 (9.0)
My twin sibling	19/619 (3.1)	2/76 (2.6)	11/410 (2.7)	6/133 (4.5)
Other	11/619 (1.8)	2/76 (2.6)	6/410 (1.5)	3/133 (2.2)

## Discussion

### Cross-comparison and Key Synthesis

This section addresses the research questions stated in the introduction through the cross-comparison of lenses.

#### Understanding Adolescents’ Perception of Own Versus Others’ Relationship With Technology

**Figure 2:**
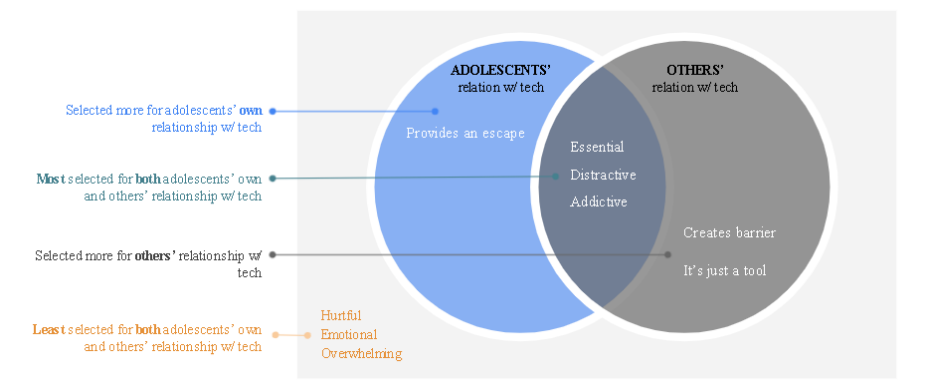
Cross-comparison between adolescents’ perceptions of their own versus others’ relationship with personal technology.

A side-by-side comparison of adolescents’ own relationship (lens 1) versus others’ relationship (lens 2) revealed some notable similarities in the way adolescents perceive their own versus others' relationship with personal technology. The Venn diagram in Figure 2 shows an overview of the alignment and mismatch between adolescents’ perceptions of their own versus others' relationship with personal technology.

We found that adolescents perceive their own as well as others' relationship with personal technology as “Essential,” Distractive,” and “Addictive.” However, there were some key differences in how adolescents perceive their own versus others' relationship with their personal technology. Adolescents associate “Provide an escape” more with their own relationship with technology. However, they attributed “It's just a tool” and “Creates Barrier” more often to others' relationship with personal technology. Moreover, adolescents did not associate “Hurtful,” “Overwhelming,” or “Emotional” with their own or others' relationship with technology.

Adolescents associated “Provide an escape” more with their own technology, whereas they associated “Creates barrier” and “It’s just a tool” more for describing others' relationship with personal technology. This difference has not been highlighted in prior research.

We were able to produce these findings by applying a multi-lens approach. A deeper understanding of the factors underlying these findings is critical and may have direct implications on technology designed for adolescents.

#### Understanding Adolescents' Perception of Their Current Relationship With Technology Versus Their Aspirations

As mentioned earlier, adolescents selected “My personal assistant” the most when asked about their aspirations of a persona for their personal technology in question 3. We further cross-compared adolescents’ aspirations (lens 3) and perceptions of current relationship with technology (lens 1). One interesting association which surfaced was that the adolescents who chose the “My best friend” persona for their personal technology, selected “Provides an escape'” as their top descriptor for their self-relationship with technology (27/148, 18.2% chose this option). Wishing for personal technology to be “My best friend” and seeing technology as something which “Provides an escape” shows critical associations that need further in-depth explorations.

In summary, our study indicates that adolescents see both commonalities and variations in their own and others' relationship with technology. Their future aspirations for personal technology vary across age and gender. We will validate our preliminary findings in a follow-up study using a larger sample size.

### Conclusions

This formative research explores adolescents' perception of relationship with personal technology. Unlike prior studies, our novel 3-lens approach is not limited to characterizing only adolescents' perception of technology as users. Instead, it further allows a comparison of adolescents’ perception of technology as users versus bystanders and from current versus aspirational perspectives. The 3-lens approach yielded findings that show both alignment and conflict in perception of self-use versus others’ use of personal technology. Our study also demonstrated variation in the perception of the youngest adolescents compared to the rest of the group as to how personal their relationship is with their personal technology.

Foundational understanding of adolescents’ relationship perception through our multi-lens approach also offers a guiding perspective to personal technology user-experience designers. This research will also empower health care professionals and youth counselors to understand aligned and conflicted perceptions and design appropriate intervention for addressing the negative implications of technology.

In the follow-up research, we will conduct a series of focus groups with adolescents of different ages. We will focus on understanding the rationale behind adolescents’ perception of the technology relationships and validate the findings from this study.
